# Disentangling the Complexity in Protein Complexes Using Complementary Isotope-Labeling and Multiple-Receiver NMR Spectroscopy

**DOI:** 10.1021/jacs.4c09176

**Published:** 2024-10-07

**Authors:** Sonja Knödlstorfer, Marco Schiavina, Maria Anna Rodella, Karin Ledolter, Robert Konrat, Roberta Pierattelli, Isabella C. Felli

**Affiliations:** † Department of Structural and Computational Biology, Max Perutz Laboratories, University of Vienna, Campus Vienna Biocenter, 5, 1030 Vienna, Austria; ‡ Vienna Doctoral School in Chemistry (DoSChem), University of Vienna, Währingerstraße 38, 1090 Vienna, Austria; § Magnetic Resonance Center and Department of Chemistry “Ugo Schiff”, University of Florence, Via Luigi Sacconi 6, 50019 Sesto Fiorentino, Florence, Italy; ∥ Christian Doppler Laboratory for High-Content Structural Biology and Biotechnology, Department of Structural and Computational Biology, Max Perutz Laboratories, University of Vienna, Campus Vienna Biocenter, 5, 1030 Vienna, Austria

## Abstract

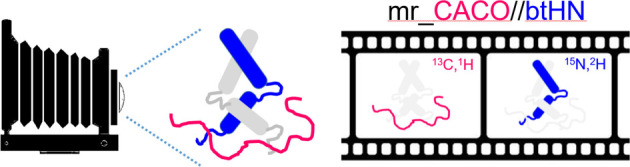

Intrinsically disordered proteins are abundant in eukaryotic systems, but they remain largely elusive pharmacological targets. NMR spectroscopy proved to be a suitable method to study these proteins and their interaction with one another or with drug candidates. Although NMR can give atomistic information about these interplays, molecular complexity due to severe spectral overlap, limited sample stability, and quantity remain an issue and hamper widespread applications. Here, we propose an approach to simultaneously map protein–protein binding sites onto two interacting partners by employing a complementary isotope-labeling strategy and a multiple receiver NMR detection scheme. With one partner being ^15^N,^2^H labeled and the interacting one being ^13^C,^1^H-labeled, we exploited proton and carbon detection to obtain clean and easily readable information. The method is illustrated with an application to the 50 kDa ternary protein complex formed between the prominent oncogenic transcription factor complex Myc/MAX and the tumor suppressor BRCA1.

Intrinsically disordered proteins (IDPs) play crucial roles in numerous cell signaling and regulatory processes and misfunction of said IDPs or intrinsically disordered regions (IDRs) is thus a leading cause for cancer and other diseases.^1−6^ The hallmark of IDPs is their dynamic interaction with and regulation of a multitude of different binding partners.^1−3,7,8^ Detailed characterization of these often transient protein interaction events is thus essential for both a deeper understanding of these fundamental biological processes and a prerequisite for subsequent drug development. However, their dynamic character and structural adaptability preclude classical (rigid) structure-based approaches and mandate the development of novel analytical techniques.^1,8−10^

Nuclear magnetic resonance spectroscopy (NMR) has matured into an indispensable tool for their investigation, as it provides high resolution information about the structural and dynamic properties of highly flexible proteins under near-physiological conditions. Nonetheless, several obstacles remain when studying IDPs by NMR. First, the poor chemical shift dispersion of amide protons resulting from largely solvent exposed backbones and their intrinsic flexibility presents a challenge. This problem can be partly overcome by extending the dimensionality of NMR experiments (recording different combinations of nuclear spins in 4D-6D correlation experiments) and harnessing the favorable relaxation properties of nuclear spins in IDPs.^11^ Second, exchange of ^1^H^N^ with water protons, in particular at physiological pH and elevated temperature, results in significant line broadening and decreased sensitivity. Alternative detection nuclei, such as ^13^C, as opposed to the amide proton, are thus quite interesting in the context of disordered systems.^10,12^ The most recent NMR hardware allows ^13^C detection with good sensitivity, also coming with other benefits such as multiple receivers which permit one to record more than one free induction decay (FID) per experimental repetition, enabling the simultaneous acquisition of different spectra, cutting down on limited experimental time, and allowing for the detection of complementary information in a single experiment.^9,13−15^

Here, we propose a novel approach that combines tailored isotope-labeling strategies, inspired by orthogonal labeling methods proposed in the literature^16−19^ with multiple receiver NMR technology^9,13,15,20^ to dissect interaction interfaces in high-molecular weight protein complexes of IDPs. In particular, the IDP component of the protein complex is investigated with ^13^C detected experiments while ^1^H–^15^N transverse-relaxation optimized (TROSY) detection schemes are used for the other protein binding partner (be it a globular, well-folded subunit, or another IDP). We illustrate the new approach with an application to the 50 kDa ternary protein complex formed between the prominent oncogenic heterodimeric transcription factor complex Myc/MAX bound to the tumor suppressor BRCA1. The heterodimeric Myc/MAX complex, constituted of the proto-oncogene Myc and MAX (Myc associated factor X),^1,21^ has been shown to exist as a four-helical bundle structure comprising two helix–loop–helix subunits.^22^

The tumor suppressor protein BRCA1 is characterized by an IDR of about 1500 residues out of 1863 in total. This IDR is flanked by two well characterized globular domains at the N- and C-terminal^1^ and harbors many proposed protein binding sites, such as the one for the transcriptionally active complex Myc/MAX.^22^ In this study, we used the following domains: the Myc/MAX interaction domain of BRCA1^219–504^; the C-terminal DNA-binding and dimerization domains of Myc^322–425^ and MAX^1–93^. To study their interaction, we opted to use a selective labeling scheme, with one binding partner (BRCA1) enriched in ^13^C and the other (MAX) enriched in ^2^H ^15^N, in complex with unlabeled Myc (^12^C,^14^N,^1^H). This labeling scheme allowed for the separate and complementary detection of MAX (via ^1^H-detected ^15^N–^1^H^N^ BEST-TROSY, btHN^23^) and BRCA1 (via ^13^C-detected CACO^24^) with multiple receiver NMR experiments (mr_NMR).

The scheme of the isotope labeling strategy and a sketch of the pulse sequence building blocks of the employed multiple receiver experiment are shown in Figure 1 (details on the pulse sequence can be found in Supplementary Figure S1, on experimental parameters used in Table S1).

**Figure 1:**
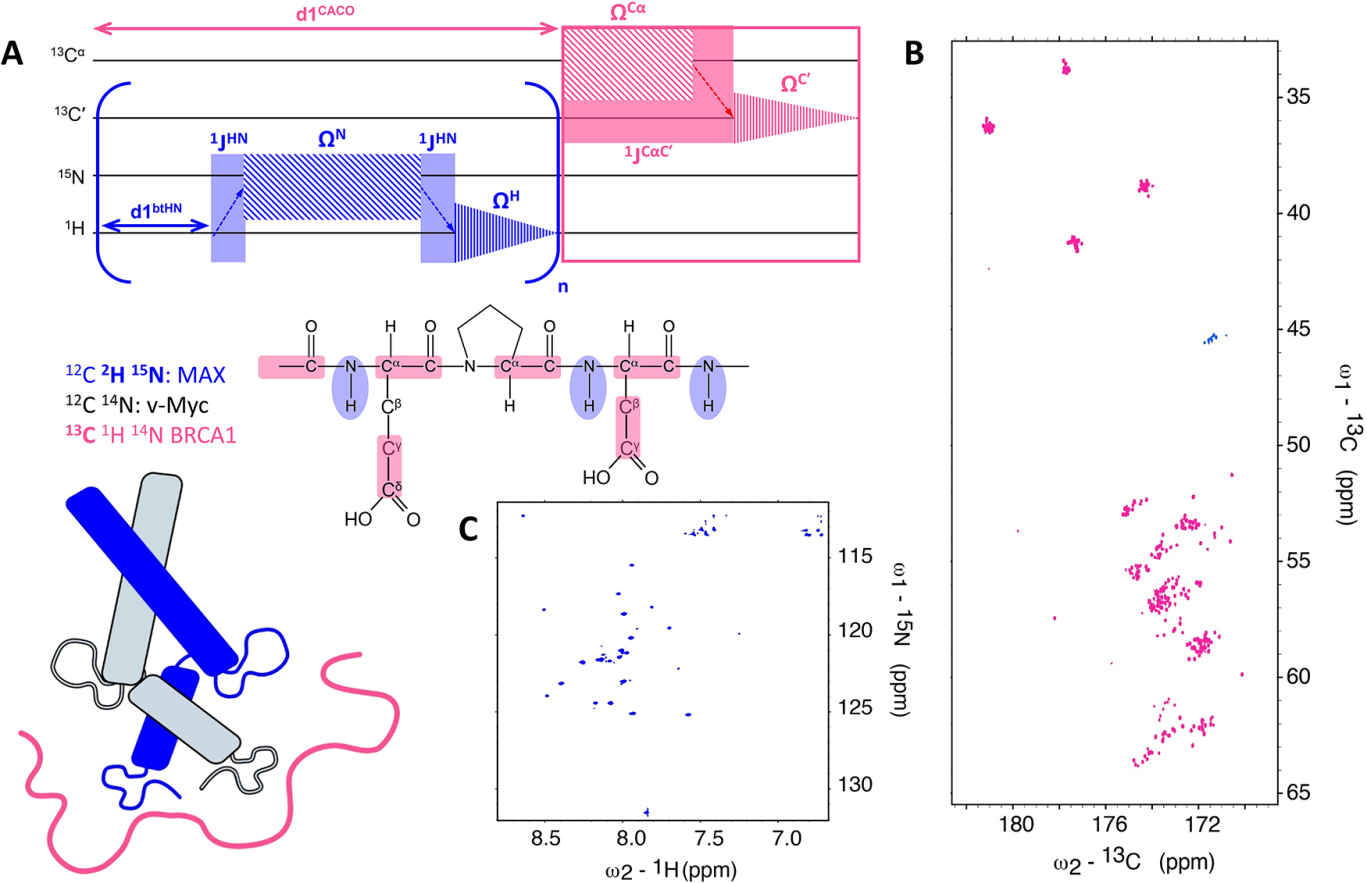
Scheme of magnetization transfer steps in the dual-receiver mr_CACO//btHN pulse sequence and of the isotopic labeling strategy employed (A). The individual magnetization transfer pathways are color-coded according to the isotope-labeling scheme (blue: ^15^N,^2^H MAX, BEST-TROSY; pink: ^13^C,^1^H BRCA1, ^13^C CACO); the type of correlations observed in the two experiments are schematically shown. More than one transient of BEST-TROSY HN can be acquired during the relaxation delay of ^13^C CACO (d1^CACO^), given the faster longitudinal relaxation of ^1^H^N^. Direct acquisition takes place in the proton dimension for the BEST-TROSY HN experiment and in the ^13^C′ dimension for ^13^C CACO. Details about the multiple receiver pulse sequence are shown in Supplementary Figure S1. Multiple receiver spectra are shown for ^13^C,^1^H BRCA1 (^13^C CACO) (B) and ^15^N–^2^H MAX (BEST-TROSY HN) (C) with the Myc/MAX heterodimer in a 1:1 ratio with BRCA1.

Overall, the flow of ^13^C magnetization, identical with the previously published ^13^C-detection experiment,^12,24^ provides information about backbone and selected side chains (Asp, Asn, Glu, Gln) as shown in Supplementary Figure S2. Most importantly, however, and due to the orthogonal labeling pattern of ^15^N,^2^H MAX and ^13^C,^1^H BRCA1, the btHN block can be inserted into the ^13^C-CACO experiment as the magnetization transfer pathways in the two experiments (btHN and ^13^C-CACO) are largely independent (Supplementary Figure S3). Although a potential relaxation interference exists due to intermolecular cross relaxation effects between ^1^H^N^ protons of MAX and ^1^H protons from BRCA1, we expect this contribution to be small and thus not affect the performance (sensitivity) of the multiple receiver experiment. More importantly, however, the selected labeling scheme allows us to avoid the ^13^C refocusing pulse that would otherwise be necessary during the chemical shift evolution in the indirect dimension of the btHN which can be repeated more than once during the relaxation delay needed for the CACO experiment; the optimal number of repeats depends on the relative longitudinal recovery times of the two experiments, a feature that should be checked before beginning a series of measurements. Also, ^15^N decoupling is not required during the (complementary) ^13^C-CACO experiment and additionally allows for longer evolution times and, as a consequence, improved spectral resolution, thus fruitfully exploiting BRCA1’s flexibility (see Supplementary Figure S1). In principle, many experimental variants can be designed, such as the one reported in Supplementary Figure 4 (mr_H^α-flip^CACO//btHN). While the two variants have similar information content, they differ with respect to sensitivity. Both versions were recorded, and a comparison of signal-to-noise (S/N) is given in Supplementary Figure S5. The performance of the dual receiver experiment is illustrated in Figure 1B,C with data obtained for the reconstituted ternary protein complex Myc/MAX/BRCA1 (1:1 adduct).

To demonstrate the applicability of the novel pulse sequence for protein interaction site mapping in a complex IDP, we performed experiments (btHN and CACO) on isolated subunits (Myc/MAX complex and BRCA1) and on the ternary protein complex Myc/MAX/BRCA1 (1:1 adduct) (mr_^13^C CACO//btHN). The experimental NMR spectra obtained with the multiple receiver (mr_NMR) versions are shown in Figure 2A,C for BRCA1 and Figure 2B,D for the Myc/MAX complex. Mapping of interaction sites in IDPs is straightforward as residues located in the binding site undergo typically significant rigidification accompanied by a reduction in signal intensity whereas unaffected residues retain their intensities. Inspection of the ratios of signal intensities of the isolated- and bound-state as a function of residue position immediately reveals the binding site (Figure S6). In the case of folded (globular) proteins, binding site residues can be identified based on chemical shift changes.

**Figure 2:**
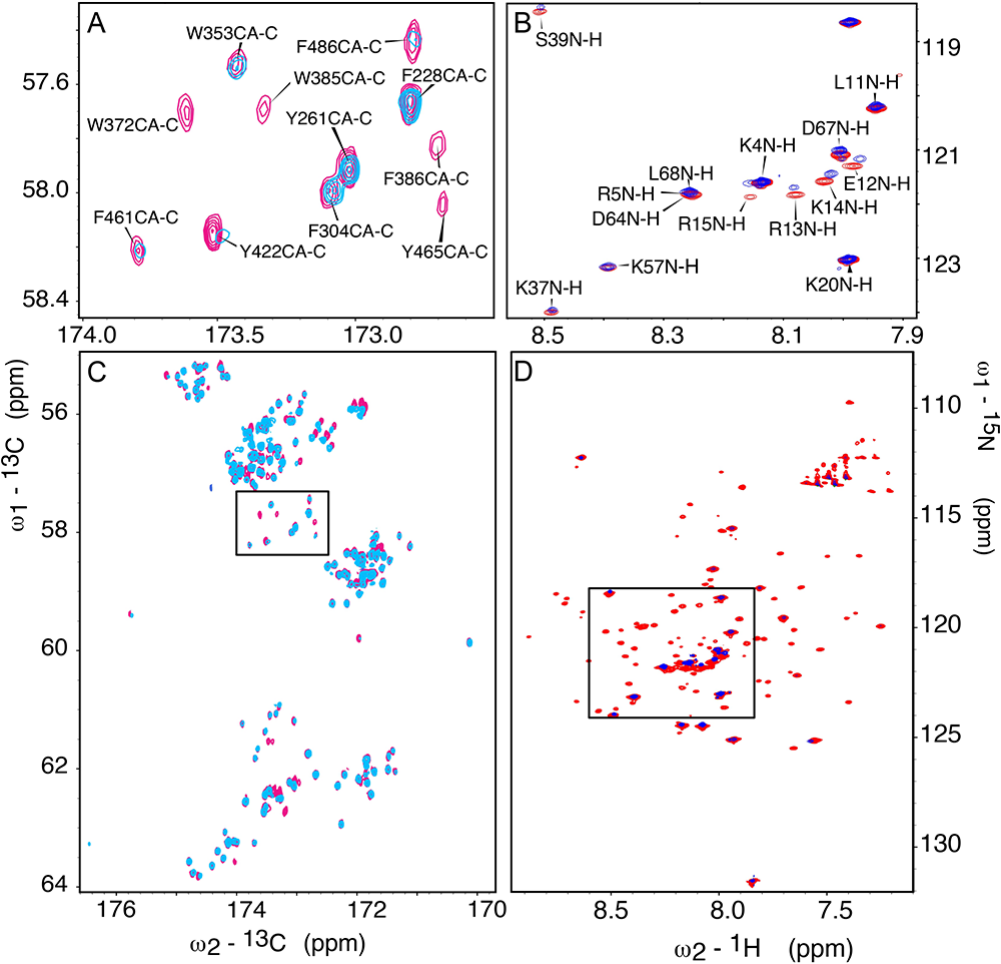
NMR binding site mapping. The CACO of BRCA1 is shown in (A, C) with a zoom in on the WFY region (A) displaying the assignment of isolated (pink) versus bound (blue) form. The *x*-axis shows the ^13^C′ chemical shifts, and the *y*-axis shows the ^13^C^α^ dimension. Residues W372, W385, F386, F461, F486, Y422, and Y465 show a decrease in intensity. Panels B and D show Myc/MAX from the point of view of MAX with and without BRCA1 1:1. Panel D displays the full Myc/MAX heterodimer isolated and bound to BRCA1 in red and blue, respectively. It should be noted that the sizable motional anisotropy of the (binary) Myc/MAX complex and the substantially increased hydrodynamic radius of the ternary Myc/MAX/BRCA1 complex leads to significant line-broadening and corresponding disappearance of peaks. Panel B shows a zoom on the flexible region, visible even with addition of BRCA1. The *x*-axis shows the proton dimension and the *y*-axis, the nitrogen chemical shifts.

For BRCA1, the isolated protein and 1:1 complex spectra were compared (Figure 2B), with a zoom in on the WFY region (Figure 2A). Assignment of Asp carboxyl side-chain signals was achieved through a combination of 2D CACO and CBCACO of BRCA1, following an already described procedure,^24^ starting from the available backbone assignment (from experiments reported in the Supplementary Table S2). Inspection of the data reveals intensity and chemical shift changes upon Myc/MAX binding for signals of BRCA1 residues in the region 370–430 (see also Supplementary Figures S6–S8). Localization of Myc/MAX epitopes was achieved by inspection of chemical shift changes in the BEST-TROSY experiment. Figure 2B shows that mainly residues in the N-terminus of MAX within the Myc/MAX complex (E12-R13-K14-R15) shift upon binding to BRCA1. It should be noted that the proton detected BEST-TROSY experiment faces challenges from the largely α-helical Myc/MAX heterodimer binding to the nearly 300 residue BRCA1 construct. Motional anisotropy and the significant increase in the effective hydrodynamic radius of the ternary complex upon BRCA1 binding lead to the disappearance of several ^15^N–^1^H signals. However, despite the very fast transverse relaxation of most of the MAX signals, residues located in the flexible regions of MAX are still detectable and resolved in the mr_NMR approach (blue peaks, Figure 2B,D).

To conclude, the NMR data show that binding of BRCA1 to Myc/MAX is promoted by interactions of the 370–430 region of BRCA1 with, at least, part of the N-terminus of MAX. The formation of the complex may be driven by electrostatic interactions involving negatively charged residues of BRCA1 (located near D390, D396, D397, D411, and D414 of BRCA1) and positively charged residues (R13-K14-R15 of MAX) adjacent to the DNA-binding basic region of Myc/MAX.^21,22^

Our results demonstrate that this novel approach may be applied to map protein interaction sites in IDP–protein complexes in a clean and straightforward way. Indeed, NMR observables are altered in many ways upon interaction in particular when investigating complex systems; the novel approach combining orthogonal labeling of the partners with multiple receivers allows us to pick up at once even subtle changes experienced by the interacting partners also in the case of highly complex systems. Furthermore, an additional advantage of the multiple receiver strategy is that one of the two acquired experiments (the one with the shorter recovery delay) comes free, since it is recorded during the recovery delay of the longer one, resulting in time savings. Although Myc/MAX’s unfavorable transverse relaxation properties, due to its pronounced motional anisotropy, precluded the observation of all backbone signals in the complex, the binding site, or at least a significant part of it, could be reliably mapped to the amino-terminal basic region of the Myc/MAX complex. Of course, we expect better performance of the approach in the case of globular (spherical) protein binding partners where motional anisotropy is less troublesome. Moreover, applications at Ultra-High fields will benefit from the significantly improved resolution for IDPs in the ^13^C dimension as already demonstrated.^25^ Encouraged by the present example, we envisage widespread applications of this approach when studying large IDP-protein complexes by combining selective isotope-labeling, using well-established precursor technology,^26−28^ with multiple receiver NMR detection schemes. The present approach could be routinely used in NMR titrations to simultaneously follow two binding partners involved in the interaction. The method could be further extended to monitor even three partners in ternary complexes through different combinations of isotopic labeling strategies and multiple receiver NMR experiments. For example, ternary protein complexes could be reconstituted with three differentially isotope-labeled protein subunits (A: ^12^C–^2^H-^13^C^1^H_3_; B: ^15^N–^2^H; C: ^13^C–^2^H) and probed by employing an interleaved multiple-receiver ^1^H^CH3^-flip ^13^C–^1^H HS(M)QC, ^1^H–^15^N BEST-TROSY, and ^13^C CACO detection scheme. Concluding, parallel acquisition of NMR spectra from different spin species, together with tailored isotope-labeling schemes, constitutes a powerful framework for the design of informative techniques to study in a radically new manner structural dynamics in macromolecular complexes.

## Supporting Information Available

Details on protein expression and purification, detailed description of the multiple receiver pulse sequences, and additional experimental information (PDF)
