# Supplementary material for: Disentangling the Complexity in Protein Complexes Using Complementary Isotope-Labeling and Multiple-Receiver NMR Spectroscopy
Source: J Am Chem Soc. 2024 Oct 7;146(41):27983–7. doi: 10.1021/jacs.4c09176 (PMC11523233; doi:10.1021/jacs.4c09176)
Supplement: Supplementary file 1 — ja4c09176_si_001.pdf [file ja4c09176_si_001.pdf]

Disentangling the Complexity in Protein Complexes using Complementary Isotope-Labeling and Multiple-Receiver NMR Spectroscopy

Sonja Knödlstorfer,[†,‡,¶] Marco Schiavina,[§,¶] Maria Anna Rodella,[§] Karin Ledolter,[†] Robert Konrat,[*,‖,†] Roberta Pierattelli,[*,§] and Isabella C. Felli[*,§]

¶These authors contributed equally to this work

†Department of Structural and Computational Biology, Max Perutz Laboratories, University of Vienna, Campus Vienna Biocenter, 5, 1030, Vienna, Austria

‡Vienna Doctoral School in Chemistry (DoSChem), University of Vienna, Währingerstraße 38, 1090, Vienna, Austria

§Magnetic Resonance Center and Department of Chemistry "Ugo Schiff", University of Florence, Via Luigi Sacconi 6, 50019 Sesto Fiorentino, Florence, Italy

‖Christian Doppler Laboratory for High-Content Structural Biology and Biotechnology, Department of Structural and Computational Biology, Max Perutz Laboratories, University of Vienna, Campus Vienna Biocenter, 5, 1030, Vienna, Austria

Table of contents

1. Sample preparation …………………………………………………………………… S2
2. NMR experiments …………………………………………………………………… S2
3. Table 1: Acquisition parameters for the mr_NMR experiments ……………………… S3
4. Table 2: Acquisition parameters used for the assignment of BRCA1 ………………… S4
5. mr_CACO//btHN pulse sequence scheme ……………………………………………… S5
6. 2D CACO spectrum highlighting backbone and side chain correlations ………………… S6
7. Comparison of spectra acquired with multiple receivers with spectra acquired separately………S7
8. mr_H$^{\alpha flip}$CACO//btHN pulse sequence scheme ………………………………… S8
9. CACO intensities comparison in the mr_CACO//btHN and in mr_H$^{\alpha flip}$CACO//btHN……… S9
10. Intensity plot of BRCA1 alone and upon addition of Myc/Max …………………………S10
11. Aspartate carboxyl side chain assignment of BRCA1 ………………………………… S11
12. Results from the aspartate side chains of BRCA1 …………………………………… S12
13. The mr_^{13}C_CACO//btHN pulse sequence …………………………………………… S13
14. The mr_H$^{\alpha flip}$CACO//btHN pulse sequence …………………………………… S21
15. References ………………………………………………………………………… S29

Sample preparation. Both ^{15}N, ^{13}C and ^{14}N, ^{13}C labeled BRCA1$^{219-504}$ were expressed using a pET11d vector and the *E. coli* strain *Rosetta pLyss*. The protein was expressed and harvested as described elsewhere[1] with 1 L minimal medium M9 containing either 1 g $^{15}NH_4Cl$ or $^{14}NH_4Cl$ and 2 g ^{13}C D-glucose. As a first purification step a HisTrap column was used.The equilibration buffer contained 25 mM Tris pH=8.0, 100 mM NaCl and 1 mM β-Mercaptoethanol, and the elution was done with a gradient with a 500 mM imidazole containing buffer, reaching up to 60 % in the gradient. Before elution the column was washed with 1 M NaCl containing Tris buffer to wash off bound DNA. The His-Tag was cleaved off with 1:50 mg/mg TEV:protein over night at 4°C, dialyzing in 25 mM Tris pH=8.0, 100 mM NaCl, 1 mM DTT buffer. A Q-Trap anion-exchange column was used as a last purification step. Here, the protein was eluted with a salt gradient up to 60 % of 1 M NaCl, 25 mM Tris pH=8.0, 1 mM DTT. The sample was concentrated and exchanged into the NMR measurement buffer which contained 20 mM Bis-Tris pH=7.0, 50 mM NaCl, 100 mM L-Arginine, 5 % glycerol, 0.2 mM sodium azide, 1 mM EDTA, 10 mM DTT, 0.1 x protease inhibitor cocktail.
^{15}N, ^{2}H Max$^{1-93}$ was produced in D_2O using pET3d vector and the *E. coli* strain *BL21pLysS*. 0.25 mL of a saturatedculture in H_2O M9 was added to 10 mL D_2O M9, which was grown over night. This culture was subsequently added to 0.5L of D_2O M9 and grown until OD_{600} of 0.6 to 0.7. Expression and purification were performed as described elsewhere[2]. Unlabeled v-Myc$^{322-425}$ was produced as previously described[2] but without isotope labeling. Both Myc and MAX were dialyzed in the NMR measurement buffer as well.

NMR experiments. The NMR experiments were all acquired at 298 K on a Bruker Avance NMR spectrometer operating at 1200.63 MHz (^{1}H), 301.90 MHz (^{13}C), and 121.66 MHz (^{15}N) frequencies equipped with a 5 mm cryogenically cooled probehead optimized for ^{13}C direct detection (cryo-TXO).
To obtain the sequence-specific assignment of the BRCA1$^{219-504}$ construct a series of 2D and 3D NMR experiments were acquired combining ^{1}H- and ^{13}C-detection. These comprise 2D CON [3], hCACO [3], 2D hCBCACO [4], 3D HNCO [5], HNcoCACB [6], HNCACB [7], hHcocaNNH [8], hNCANNH [9] and hCBCACON [10] (for ^{1}H detected experiments the BEST- TROSY variants were selected[11,12]).
The mr CACO//btHN experiment was designed to monitor the interaction of the BRCA1 protein with the Myc/MAX complex using a specific isotopic labeling (as in Sample preparation) needed for the protein assignment.
Standard pulse lengths and carrier frequencies used for triple resonance experiments were used for all the NMR experiments. The ^{1}H carrier was placed at 4.7 ppm for non-selective hard pulses or at 8.3 ppm for band-selective pulses on the amide proton region. The center of ^{13}C band-selective pulses were set at 176.1 ppm, 55.9 ppm and 45.4 ppm for C', C^{α}, C^{ali} regions. ^{15}N pulses were given at 123 ppm (for ^{13}C detected experiments) or 118.0 ppm (for ^{1}H detected experiments). SURBOP90 and SURBOP180 [13] shapes of durations of 333 µs, 15 kHz of field strength, were used for ^{13}C band-selective $\pi/2$ and π flip angle pulses respectively except for the adiabatic π pulse to invert both C' and C^{α} (smoothed Chirp [14] 500 µs, 20 % smoothing,100 kHz sweep width, 12.6 kHz field strength). Composite pulse decoupling was applied on ^{1}H (Garp-4 [15]) with an RF field strength of 3.3. kHz. ^{15}N decoupling was achieved with an RF field strength of 0.89 kHz through the p5m4 composite pulse decoupling scheme that exploits a series of adiabatic Chirp inversion pulses of 8 kHz sweep, 4 ms length, 20 % smoothing and 4096 data points for digitization (Crp8,4.0,20.4). ^{13}C homonuclear decoupling for the hCBCACON, mr_^{13}C_CACO//btHN, mr_$H^{\alpha\ flip}$CACO//btHN, CON, hCACO and hCBCACO experiments was achieved through IPAP virtual decoupling[3].
The remaining experimental parameters (type of experiment, spectral width, acquired data points and acquisition times, number of scans, inter-scan delays, experimental time) are reported in Supplementary table 1 for the multiple receiver experiments, Supplementary table 2 for 2D and 3D assignment experiments.
All acquired spectra were processed and analyzed either with Bruker TopSpin 4.0.8 or NMRPipe 11.5[16]; Poky[17] and Python 3.9.12 were used for residue assignment and data analysis.

Supplementary Table 1: Acquisition parameters for the mr_NMR experiments acquired on the ternary complex. For each row the parameters refer to the underlined bold experiment.

<table>
<tr><th></th><th>Scans</th><th>Inter scan delay (s)</th><th colspan="2">Spectral Width (Hz) and maximal evolution times</th><th colspan="2">Dimension of acquired data</th></tr>
<tr><td>2D Experiments</td><td colspan="2"></td><td>F1</td><td>F2</td><td>F1</td><td>F2</td></tr>
<tr><td>mr_^{13}C_CACO//<u>btHN</u></td><td>64</td><td rowspan="2">2.01</td><td>3413 (^{15}N)
92 ms</td><td>17857 (^{1}H)
230 ms</td><td>628</td><td>8192</td></tr>
<tr><td>mr_<u>^{13}C_CACO</u>//btHN</td><td>16</td><td>11494 (^{13}C)
28 ms</td><td>6579 (^{13}C)
156 ms</td><td>628</td><td>2048</td></tr>
<tr><td>mr_H$^{\alpha\ flip}$CACO//<u>btHN</u></td><td>128</td><td rowspan="2">1.22</td><td>3412 (^{15}N)
70 ms</td><td>17857 (^{1}H)
230 ms</td><td>480</td><td>8192</td></tr>
<tr><td>mr_<u>H$^{\alpha\ flip}$CACO</u>//btHN</td><td>32</td><td>8454 (^{13}C)
28 ms</td><td>6579 (^{13}C)
155 ms</td><td>480</td><td>2048</td></tr>
</table>

Supplementary Table 2: Acquisition parameters used for the assignment of BRCA1.

	Scans	Inter Scan delay (s)	Spectral Width (Hz) and maximal evolution times		Dimension of acquired data	
2D Experiments			F1	F2	F1	F2
CON	8	2.15	4878 (^{15}N) 150 ms	9090 (^{13}C) 150 ms	1462	2752
hCACO	8	1.15	11494 (^{13}C) 27 ms	6579 (^{13}C) 150 ms	628	1972
hCBCACO	8	1.15	18182 (^{13}C) 23 ms	6579 (^{13}C) 150 ms	826	1972

3D Experiments			F1	F2	F3	F1	F2	F3
BT HNCO	4	0.32	1815 (^{13}C) 35 ms	2801 (^{15}N) 46 ms	19230 (^{1}H) 213 ms	128	256	8192
BT HNcoCACB	8	0.32	18182 (^{13}C) 4 ms	2801 (^{15}N) 43 ms	19230 (^{1}H) 213 ms	144	240	8192
BT HNCACB	8	0.32	18182 (^{13}C) 5 ms	2801 (^{15}N) 46 ms	19230 (^{1}H) 213 ms	192	256	8192
BT hNcocaNNH	32	0.42	2801 (^{15}N) 23 ms	2801 (^{15}N) 23 ms	19230 (^{1}H) 213 ms	128	128	8192
BT hNCANNH	32	0.42	2801 (^{15}N) 18 ms	2801 (^{15}N) 18 ms	19230 (^{1}H) 213 ms	100	100	8192
hCBCACON	4	1	18182 (^{13}C) 6 ms	4273 (^{15}N) 15 ms	9615 (^{13}C) 92 ms	224	128	1772

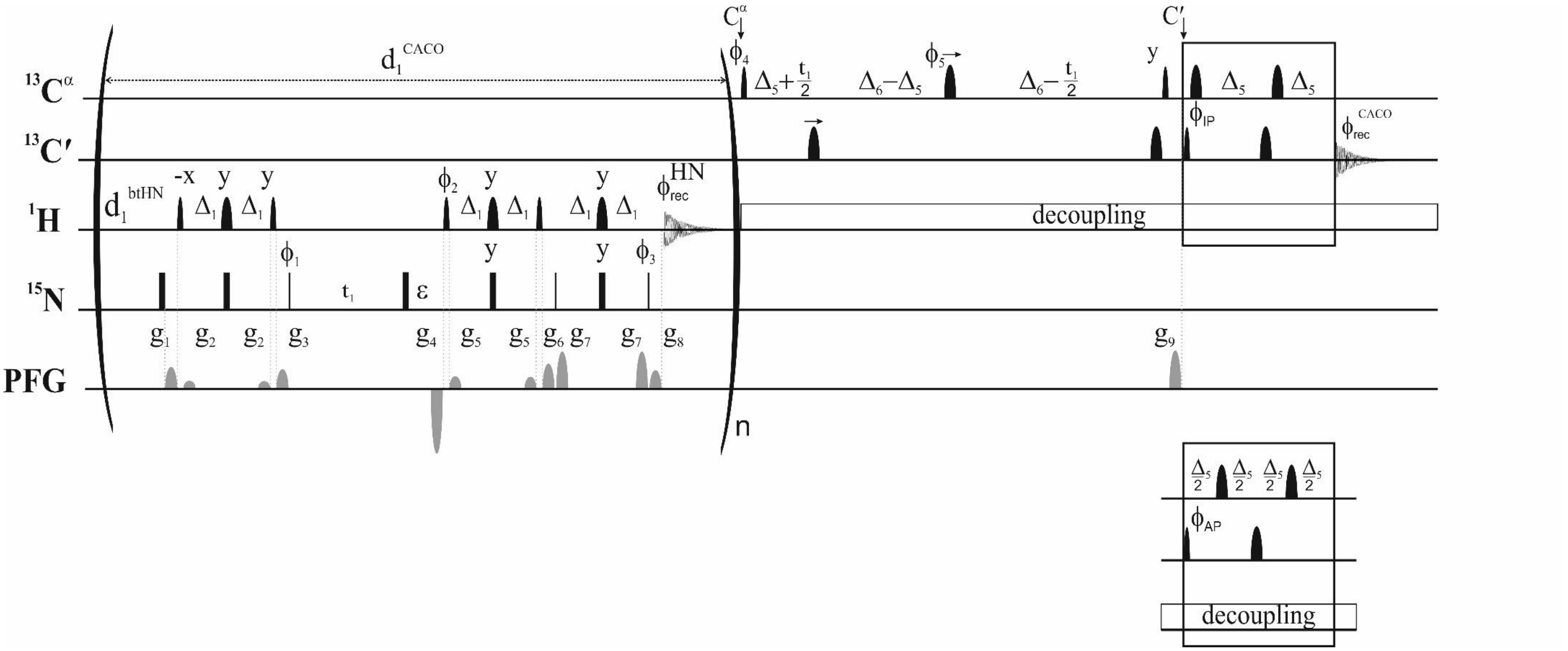

Supplementary Figure 1. Scheme of mr_^{13}C_CACO//btHN pulse sequence. Narrow and wide black rectangles represent $\pi/2$ and π non-selective pulses; narrow and wide black bell shapes represent $\pi/2$ and π band-selective pulses. The following phase cycling was employed for the mr_CACO//btHN: ϕ_1= x, -x; ϕ_2= y; ϕ_3= y; ϕ_4= x, -x; ϕ_5= 4(x), 4(y); ϕ_{IP}= 2(x), 2(-x); ϕ_{AP}= 2(-y), 2(y), $\phi_{recbtHN}$= x, -x, and $\phi_{rechCACO}$= x, -x, -x, x, -x, x, x, -x. Quadrature detection in the indirect dimension was achieved through the echo-antiecho approach exploiting phases ϕ_1, ϕ_2, ϕ_3, $\phi_{recbtHN}$ and gradients g_4, g_6 nd g_8 for the BEST-TROSY and through States-TPPI incrementing ϕ_4 for CACO. The length of the delays was: d_{1hCACO}= 2.0 s; d_{1btHN}= 0.7 s; Δ_1= 2.7 ms; Δ_5= 4.5 ms; Δ_6= 13.3 ms; ε=$t_1(0)$. Gradients length and amplitudes were: g_1 = 1.00 ms 27%, g_2 = 0.60 ms 2%, g_3 = 1.00 ms 21%, g_4 = 1.00 ms -80%, g_5 = 0.25 ms 5%, g_6 = 1.00 ms 30%, g_7 = 1.00 ms 45%, g_8 = 1.00 ms 30.13%, g_9 = 1.00 ms 50%. The pulse sequence elements reported in the boxes represent the two variants to acquire the in-phase (IP) and antiphase (AP) components of carbonyl signals needed to achieve ^{13}C homonuclear decoupling through the IPAP approach.

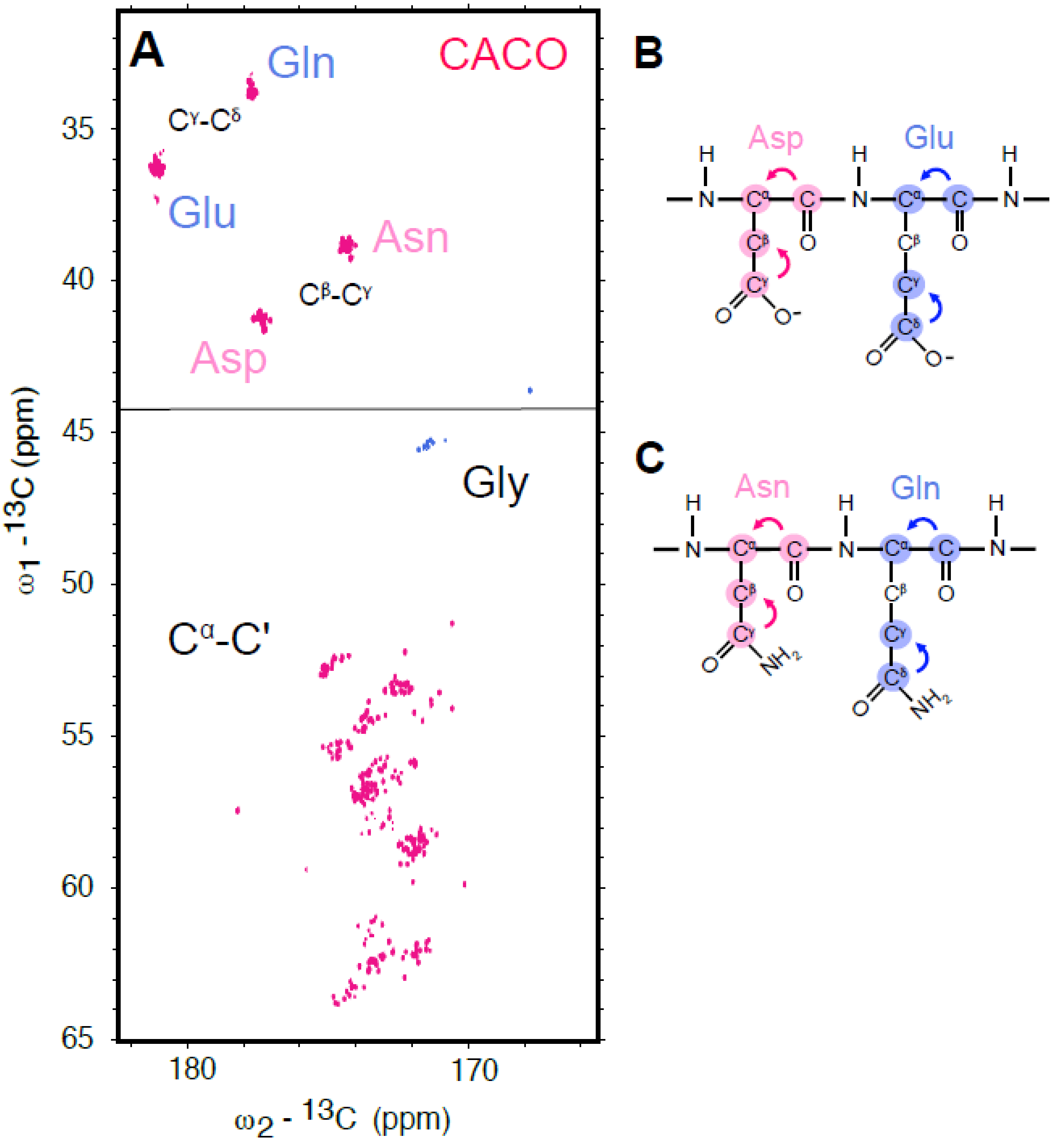

Supplementary Figure 2. The 2D CACO spectrum acquired with the mr_CACO//btHN experiment on BRCA1 is shown in panel A to highlight the different spectral regions where backbone and side chains resonances are observed. Panels B and C show the correlations expected for Asp, Asn, Glu and Gln side chains' and for all backbone resonances.

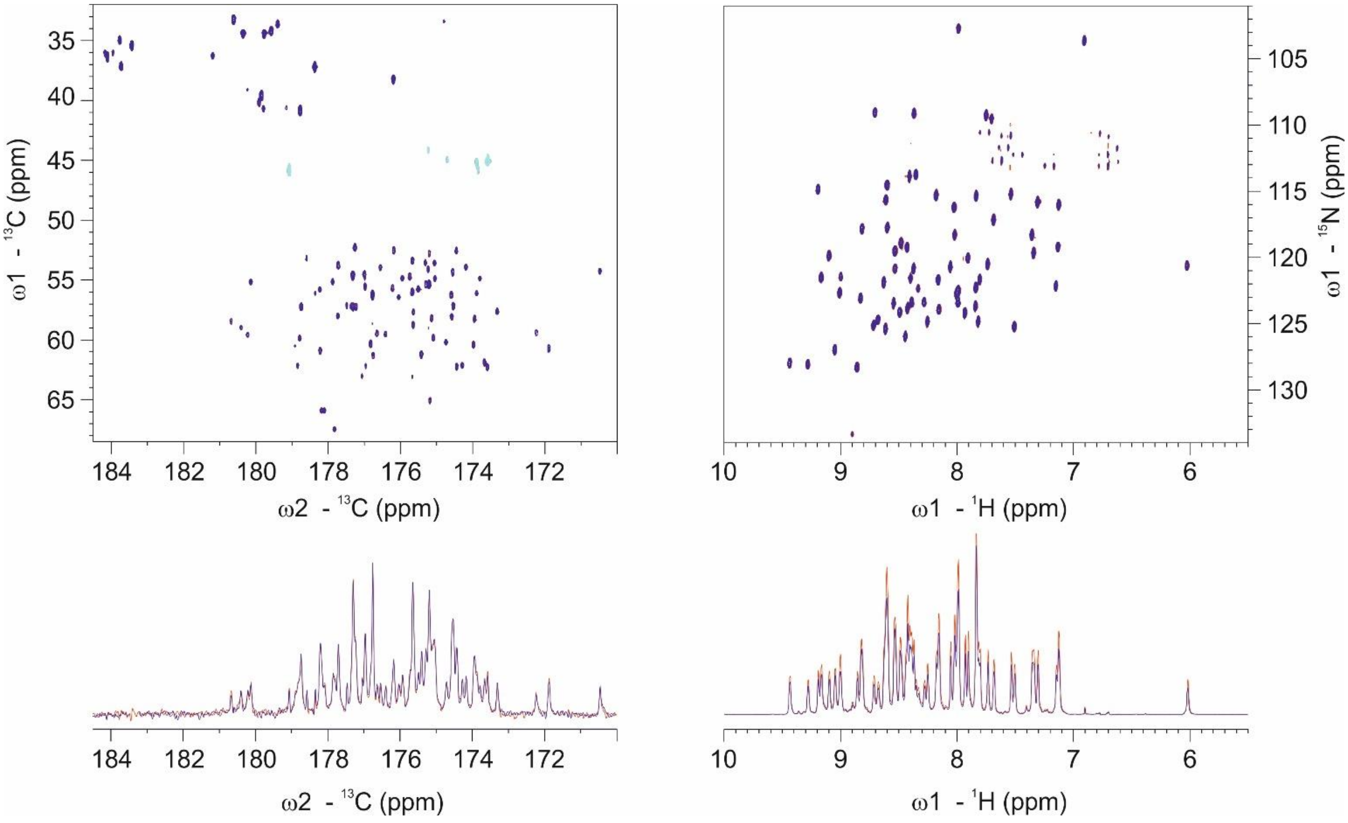

Supplementary Figure 3. Experimental comparison of the performance of the mr_CACO//btHN experiment with the two experiments acquired separately on ubiquitin. The two spectra obtained with the mr_CACO//btHN experiment are reported on the top (CACO on the left, btHN on the right) and are overlayed with the two spectra acquired independently, one after the other. It can be noted that differences are hardly noticeable. For completeness the horizontal projections of each 2D spectrum are compared in the bottom panels; the traces of the spectra acquired with the mr_CACO//btHN experiment are reported in blue while those of the two spectra acquired independently are reported in red. For the CACO experiment there is essentially no compromise when acquiring the two spectra simultaneously with the mr_CACO//btHN experiment while for the btHN a modest reduction in sensitivity (about 10 %) is observed. This comparison was performed on a standard ubiquitin sample used for the experimental set-up (1.0 mM ubiquitin in 50 mM sodium phosphate pH 6.5 T = 298 K) using the most simple mr_CACO//btHN variant with one btHN repetition (n=1).
Including an additional btHN repetition does not influence the results for the CACO experiment and provides a significant increase in sensitivity of the btHN itself. Similar results are obtained with the mr_H$^{\alpha\text{-flip}}$CACO//btHN variant of the pulse sequence described in Supplementary Figure 4 with the only difference that some compromise is observed for the CACO spectrum when acquiring it simultaneously to the btHN. However, this is more pronounced for the globular domain (about 40%) while it is moderate for the flexible regions (about 10%). Including an additional btHN repetition, does not influence the results for the CACO spectrum and provides an increase in sensitivity of the btHN itself.

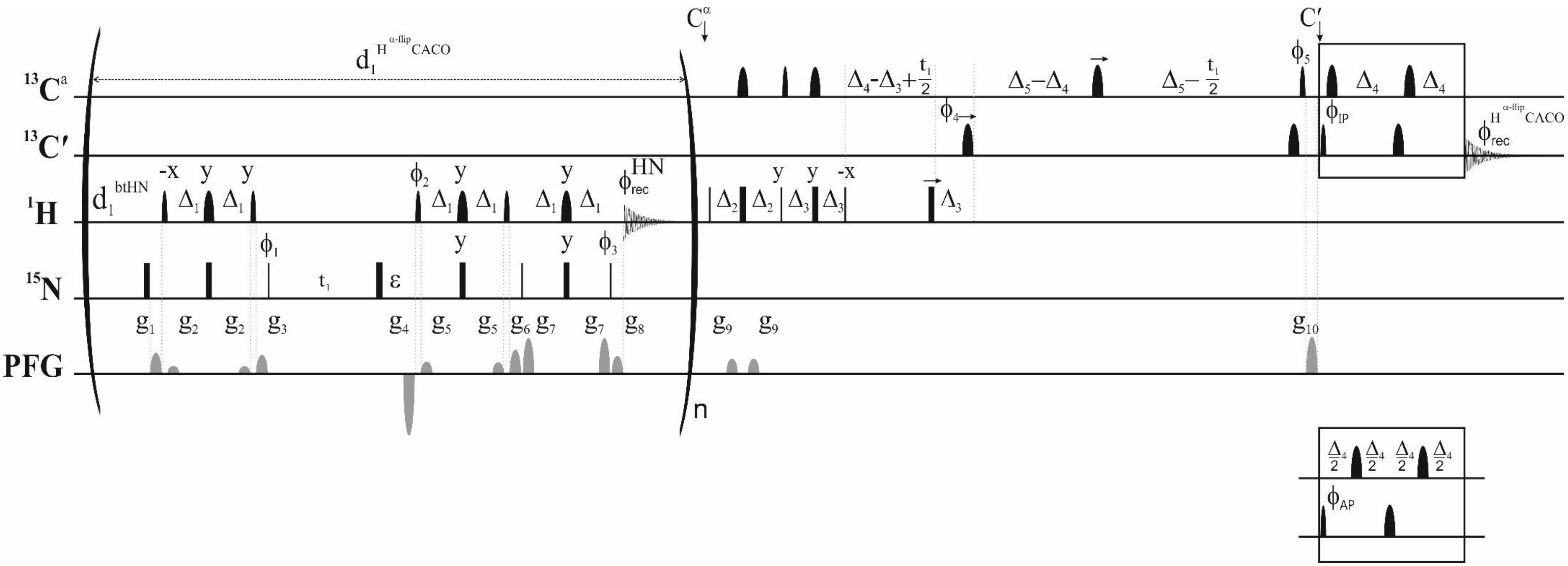

Supplementary Figure 4. Scheme of mr_H$^{\alpha flip}$CACO//btHN pulse sequence. Narrow and wide black rectangles represent $\pi/2$ and π non-selective pulses; narrow and wide black bell shapes represent $\pi/2$ and π band-selective pulses. The following phase cycling was employed for the mr_H$^{\alpha flip}$CACO//btHN: ϕ_1= x, -x; ϕ_2= y; ϕ_3= y; ϕ_4= 4(x), 4(-x); ϕ_5= x, -x; ϕ_{IP}= 2(x), 2(-x); ϕ_{AP}= 2(y), 2(-y), $\phi_{recbtHN}$= x, -x, x, -x and $\phi_{rechCACO}$= x, -x, -x, x. Quadrature detection in the indirect dimension was achieved through the echo-antiecho approach exploiting phase ϕ_1, ϕ_2, ϕ_3, $\phi_{recbtHN}$ and gradients g_4, g_6, g_8 for the BEST-TROSY and through States-TPPI incrementing ϕ_5 for H$^{\alpha flip}$CACO. The length of the delays was: d_{1hCACO}= 1.6 s; d_{1btHN} = 0.3 s; Δ_1= 2.7 ms; Δ_2= 1.8 ms; Δ_3= 1.1 ms; Δ_4= 4.5 ms; Δ_5= 14.2 ms; $\varepsilon = t_1(0)$. Gradients length and amplitudes were: g_1 = 1.00 ms 31%, g_2 = 0.60 ms 2%, g_3 = 1.00 ms 21%, g_4 = 1.00 ms -80%, g_5 = 0.25 ms 5%, g_6 = 1.00 ms 30%, g_7 = 1.00 ms 45%, g_8 = 1.00 ms 30.13%, g_9 = 1.00 ms 27%, g_{10} = 1.00 ms 50%. The pulse sequence elements reported in the boxes represent the two variants to acquire the in-phase (IP) and antiphase (AP) components of carbonyl signals needed to achieve ^{13}C homonuclear decoupling through the IPAP approach.

\

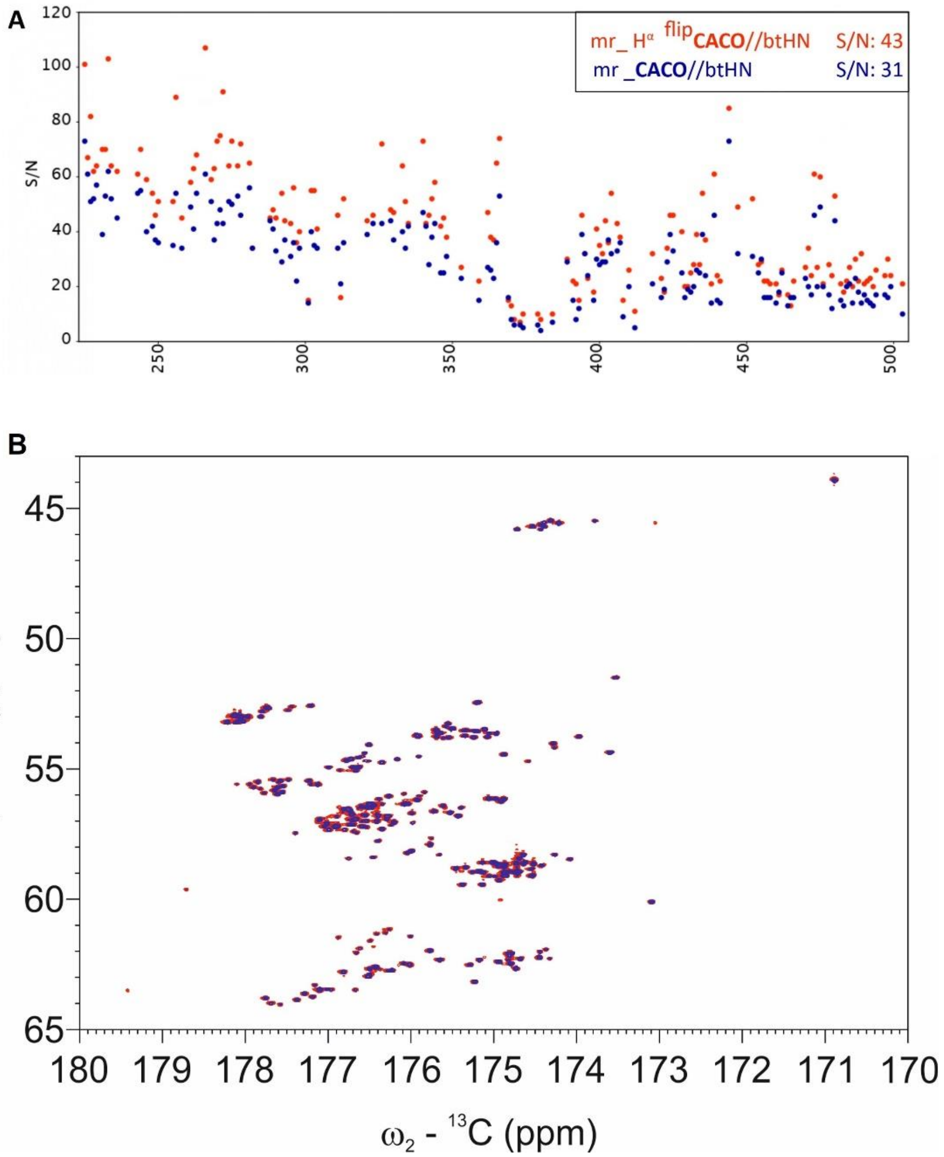

Supplementary Figure 5. Comparison of the signal to noise (S/N) ratio between the mr_$H^{\alpha flip}$CACO//btHN (red dots) and the mr_^{13}C_CACO//btHN (blue dots). Panel A reports the S/N of the cross peaks observed in the two spectra Vs the residue number; an overlay of the two spectra is reported in panel B with the same color coding.

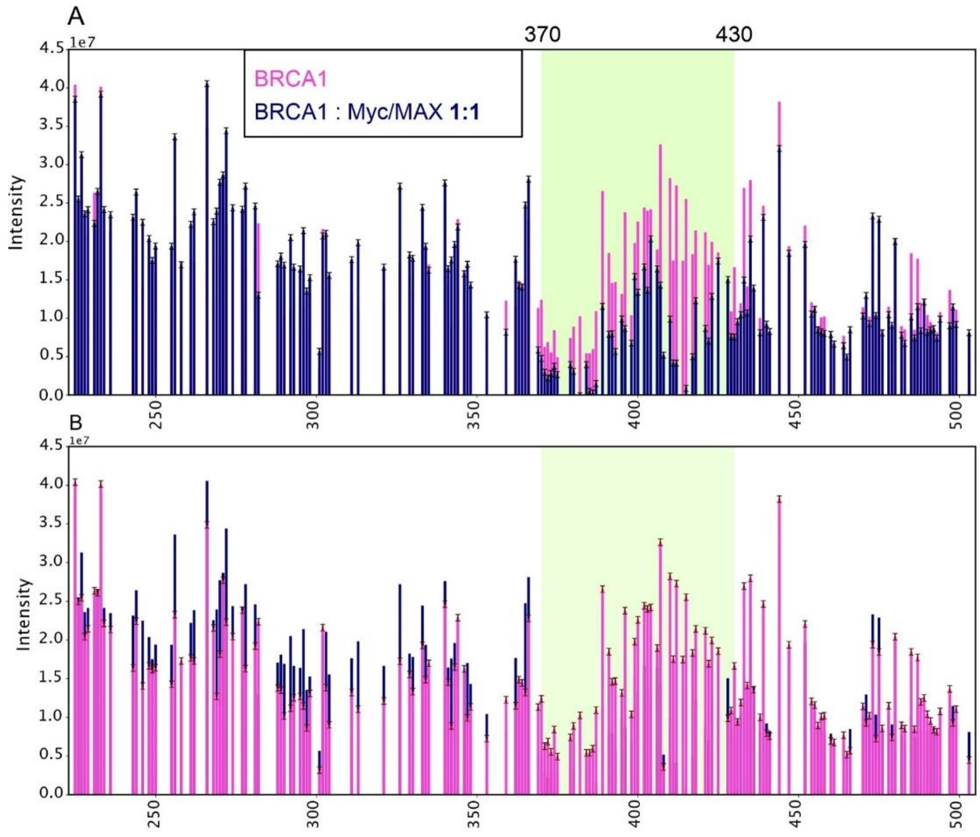

Supplementary Figure 6. Intensity plot of BRCA1$^{219\text{-}504}$ alone (pink) and with MycMAX added 1:1 (blue) are reported against the residue number (Panels A and B). The most affected region (residues 370-430) is highlighted in yellow/green. Differential effects on the residual dynamics upon complex formation are observed. However at this stage we are interested in mapping the binding site location, which corresponds to the tract of the protein that becomes less flexible (more rigid) when interacting with the more folded partner and thus experiences the most pronounced reduction in the intensity of cross peaks due to binding. Cross peaks in overlap have not been considered; error bars were estimated using Poky[17]. Intensities needed to be rescaled since experiments were acquired on different samples; they were rendered qualitatively comparable by dividing the five maximum values of BRCA1 and BRCA1/Myc/MAX and aligning all other residues through the resulting factor.

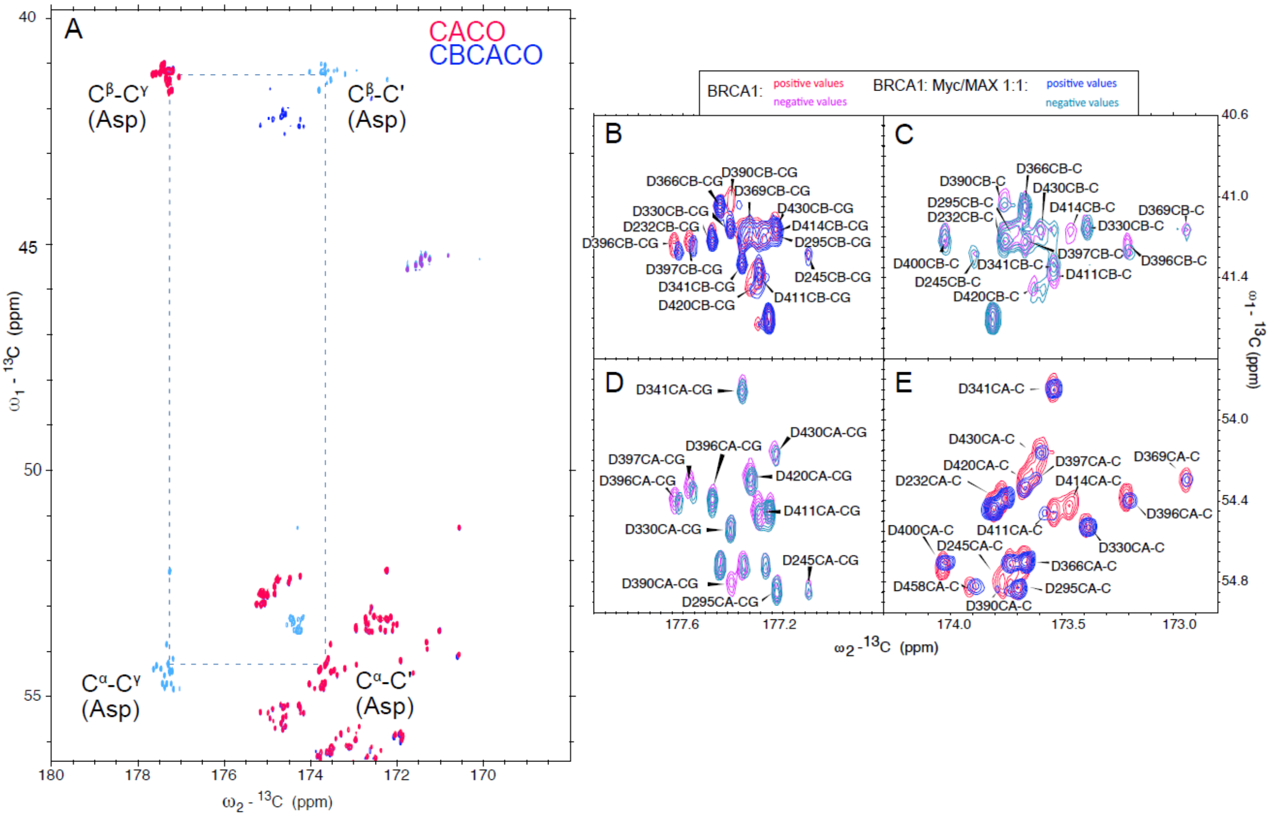

Supplementary Figure 7. A scheme of the aspartate carboxyl side chain assignment strategy of BRCA1. Panel A) explains the assignment set-up using the data acquired on BRCA1 as an example. Through the connection between the four peaks observed in specific spectral regions using the CACO (pink) and the CBCACO (blue) one can assign in a sequence specific manner the side chain resonances of aspartates. With this procedure 14 out of 19 aspartate side chains were assigned. Panels B-E) show a close-up of the C^{γ}-C^{β} (upper left, B), C^{γ}-C^{α} (lower left, D) as well as the correlations of backbone C′ with C^{α} or C^{β}, lower (E) and upper right (C), respectively in CBCACO spectra. The x-axis shows the carbonyl/carboxyl carbon chemical shifts and the y-axis the C^{α}/C^{β} dimension. As shown in the legend both BRCA1 (pink, lilac) and BRCA1:Myc/MAX (blue, cyan) forms are shown, with their positive (C^{γ}-C^{β}, C′-C^{α}) and negative (C^{γ}-C^{α}, C′-C^{β}) cross peaks. The side chain signals of the aspartates are well dispersed facilitating their assignment and analysis.

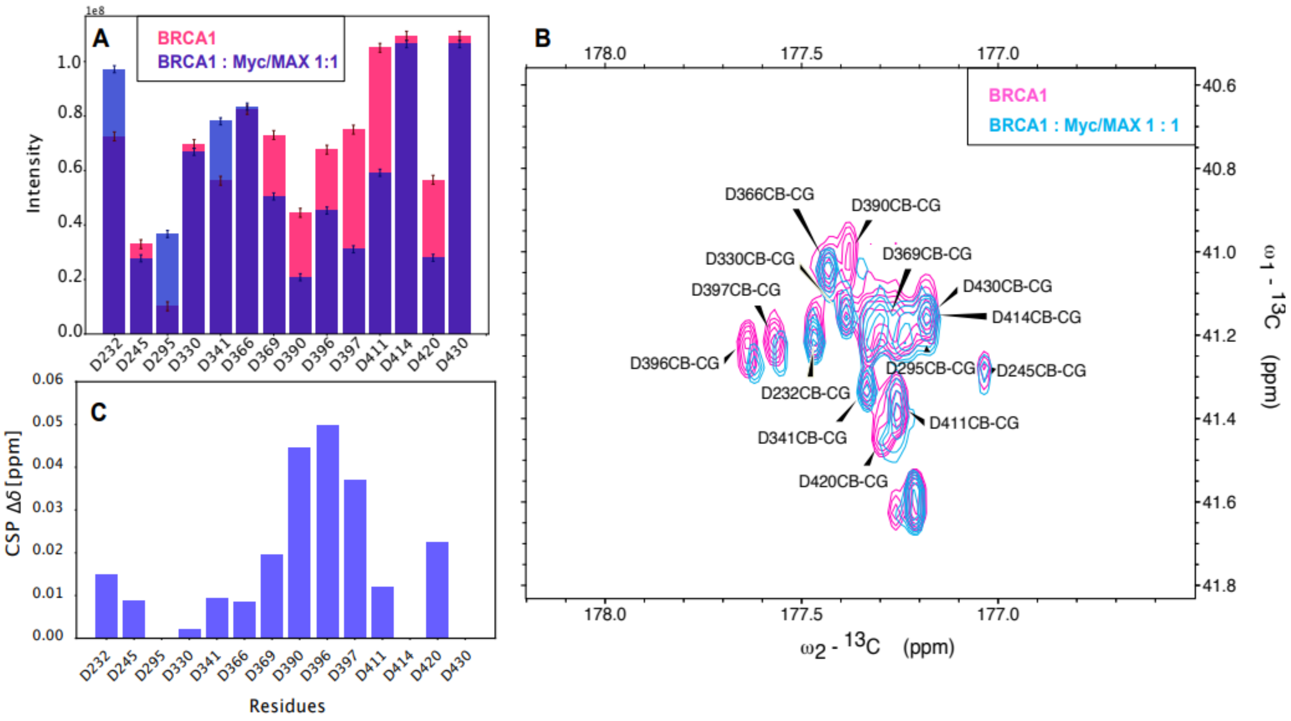

Supplementary Figure 8. Summary of the results for the aspartate side chains of BRCA1. Panel A) shows an intensity plot done as in Supplementary Figure 6 (the scaling factor was extracted from the C^{α}-C' correlations in CBCACO spectra, which contain the aspartate C^{β}-C^{γ} correlations used for this analysis, as shown in B). Pink refers to BRCA1 and blue to the BRCA1/Myc/MAX complex; the opacity of the bars reported in front allows to see also those reported in the back. A similar pattern as for the backbone in Supplementary Figure S6 can be observed. As an example a portion of the spectra used to produce panel A are shown in panel B using the same colour code, also explained in the legend (14 out of 19 aspartates were assigned). Additionally, panel C) shows the chemical shift perturbation (CSP) of these residues. The y-axis shows the CSP in ppm and the x-axis the residue number. The CSP was calculated using $\Delta\delta = \sqrt{\Delta\delta C^{\beta^2} + \Delta\delta C^{\gamma^2}}$ with $\Delta\delta C^{\beta}$ being the chemical shift changes for C^{β} and $\Delta\delta C^{\gamma}$ for the side chain carboxyl group carbon measured in ppm.

The mr_^{13}C_CACO//btHN pulse sequence

```
;ut_bthn_caco.ms.t1
;avance-version (18/09/17)

;Dataset 1 (F1)
;best-TROSY
;2D H-1/X correlation via TROSY
;   using sensitivity improvement
;phase sensitive using Echo/Antiecho gradient selection
;using f3 - channel
;uncompensated version d25=d26
;with additional 180degree pulse on N-15
;(use parameterset B_TROSYETF3GPSI)
;
;A. Favier & B. Brutscher, J. Biomol. NMR 49, 9-15 (2011)
;(E. Lescop, P. Schanda & B. Brutscher,
;   J. Magn. Reson.  187 163-169 (2007))
;(T. Schulte-Herbrueggen & O.W. Sorensen, J. Magn. Reson. 144,
;   123 - 128 (2000))
;
;$CLASS=HighRes
;$DIM=2D
;$TYPE=
;$SUBTYPE=
;$COMMENT=

;Dataset 2 (F2)
;CON
;2D sequence with
;   13C detected correlation for triple resonance using
;      inept transfer steps
;
;      F1(C=O) -> F3(N,t1) -> F1(C=O,t2)
;
;on/off resonance 13C pulses using shaped pulses
;phase sensitive (t1)
;using IPAP scheme for virtual decoupling
;(use parameterset C_CON_IASQ)
;
;W. Bermel, I. Bertini,L. Duma, I.C. Felli, L. Emsley,  R. Pierattelli,
;   P.R. Vasos, Angew. Chem. Int. Ed. 44, 3089-3092 (2005)
;(L. Duma, S. Hediger, A. Lesage & L. Emsley,
;   J. Magn. Reson. 164, 187-195 (2003) )
;
;$CLASS=HighRes
;$DIM=2D
;$TYPE=
;$SUBTYPE=
;$COMMENT=


prosol relations=<triple>


#include <Avance.incl>
#include <Delay.incl>
#include <Grad.incl>


define list<gradient> EA3 = { 1.0000 0.8750 }
define list<gradient> EA5 = { 0.6667 1.0000 }
```

```
define list<gradient> EA7 = { 1.0000 0.6595 }

"p2=p1*2"
"p22=p21*2"

"p29=250" ;from bthn

"d11=30m"
"d12=20u"


"d22=4.5m" ;from c_caco_ia
"d27=13.3m"

"d25=2.7m" ;from bthn
"d26=2.7m" ;from bthn

"d31=3u" ;from bthn
"d32=d22+4u";from c_caco_ctia
"d33=d27-p14*1.5";from c_caco_ctia

"d1=(d60+d59)*2+d58" ;d60=recycle bthn d59=aq bthn d58=aq caco

"in31=inf1/2"  ;from bthn
"in32=in2/2"   ;from c_caco_ia
"in33=in2/2"    ;from c_caco_ctia



#   ifdef CALC_SP
"p42=(bwfac26/(cnst55*cnst52*bf1))*1000000"
"spw26=plw1*pow((p1*totrot26)/(p42*90.0*integfac26),2)"
"spoal26=0.5"

"p43=(bwfac28/(cnst55*cnst53*bf1))*1000000"
"spw28=plw1*pow((p1*totrot28)/(p43*90.0*integfac28),2)"
"spw29=plw1*pow((p1*totrot29)/(p43*90.0*integfac29),2)"
"spoal28=1"
"spoal29=0"
#   endif /*CALC_SP*/



"DELTA=d31*2+p21*4/PI"
"DELTA1=d26-p19-d16-larger(p22,p42)/2"
"DELTA10=d25-p29-d16-larger(p22,p42)/2-p43*cnst43"
"DELTA11=d26-p16-d16-larger(p22,p42)/2"
"DELTA12=de+4u"

"DELTA2=d27-d22-p14*1.5"



;"FACTOR1=d28*10000000/l3" ;from c_caco_ia
;"INCR1=FACTOR1/10000000" ;from c_caco_ia

;"if ( INCR1 > in32 ) { in33 = 0; } else { in33=in32-INCR1; }" ;from c_caco_ia
;"if ( INCR1 > in32 ) { in28 = in32; } else { in28 = INCR1; }" ;from c_caco_ia

"l0=1"
```

```
"l3=td1/2"


"spoffs2=0"
"spoffs3=0"
"spoffs8=0"
"spoffs9=0"
"spoffs5=bf2*((cnst22-cnst21)/1000000)"
"spoffs20=bf2*((cnst21-cnst22)/1000000)"


"spoffs26=bf1*(cnst54/1000000)-o1"
"spoffs28=bf1*(cnst54/1000000)-o1"
"spoffs29=bf1*(cnst54/1000000)-o1"
"spoffs13=bf2*((cnst26-cnst21)/1000000)"



1 4u ze1 do:f1
  4u ze2
  d1

2 d11 do:f1 do:f3
  3m
3 12m do:f1 do:f3
4 6m do:f1 do:f3
5 6m do:f1 do:f3 ;d1

; bthn

6 d11 do:f1 do:f3
  d60

  20u fq=cnst21(bf ppm):f2
  d12 pl1:f1 pl3:f3
  50u UNBLKGRAD
  (p22 ph11):f3
  p16:gp2


  (p43:sp28 ph13)
  p19:gp11
  d16
  DELTA1
  (center (p42:sp26 ph12) (p22 ph11):f3 )
  DELTA1
  p19:gp11
  d16
  (p43:sp29 ph12):f1

  p16:gp12
  d16
  4u

  (p21 ph15):f3
  d31

  ;(p8:sp13 ph11):f2

  d31
  (p22 ph11):f3
  DELTA
```

```
  p16:gp13*EA3
  d16

  (p43:sp29 ph16)
  p29:gp14
  d16
  DELTA10
  (center (p42:sp26 ph12) (p22 ph12):f3 )
  DELTA10
  p29:gp14
  d16
  (p43:sp28 ph11)

  p16:gp15*EA5
  d16
  DELTA12

  (p21 ph11):f3
  p16:gp16
  d16
  DELTA11
  (center (p42:sp26 ph12) (p22 ph12):f3 )
  DELTA11
  p16:gp16
  d16 pl1:f1
  (p21 ph17:r):f3

  p16:gp17*EA7
  d16

  4u BLKGRAD

  goscnp1 ph31

  lo to 6 times 2

;c_caco

  8u do:f3 do:f1
  50u UNBLKGRAD
  20u pl19:f1
  20u pl16:f3
  20u fq=cnst22(bf ppm):f2
  d12 cpds1:f1 cpd3:f3

  (p13:sp2 ph3):f2
  d32
  (p14:sp20 ph1):f2
  DELTA2
  (p14:sp3 ph4):f2
  d33
  (p14:sp20 ph1):f2
  4u
  (p13:sp8 ph2):f2

  p16:gp1
  d16 fq=cnst21(bf ppm):f2


  if "l0 %2 == 1"
     {
```

```
      (p13:sp2 ph5):f2
      4u
      (p14:sp5 ph1):f2
      d22
      (p14:sp3 ph1):f2
      4u
      (p14:sp5 ph1):f2
      d22 BLKGRAD ;do:f1
      }
   else
      {
      (p13:sp2 ph6):f2
      4u
      d22*0.5
      (p14:sp5 ph1):f2
      d22*0.5
      (p14:sp3 ph1):f2
      4u
      d22*0.5
      (p14:sp5 ph1):f2
      d22*0.5 BLKGRAD ;do:f1
      }

   go2=2 ph30

   d11 do:f1 do:f3 wr2 #1 if2 #1 zd2

;exp_f2 ipap
   3m iu0
   lo to 3 times 2

   3m wr1 #0 if1 #0 zd1

;exp_f1 phase
   3m ip16*2
   3m ip17*2
   3m ip15*2
   3m ip31*2

;exp_f1 gradients
   3m igrad EA3
   3m igrad EA5
   3m igrad EA7

;exp_f2 phase
   3m ip3
   lo to 4 times 2

;exp_f1 delay
   3m id31
;exp_f2 delay
   3m id32
   3m dd33
   lo to 5 times l3

exit


ph1=0
ph2=1
ph3=0 2
ph4=0 0 0 0 1 1 1 1
```

```
ph5=0 0 2 2
ph6=3 3 1 1

ph11=0
ph12=1
ph13=2
ph14=3
ph15=0 2
ph16=1
ph17=1
ph21=2


ph30=0 2 2 0 2 0 0 2
ph31=0 2 0 2 0 2 0 2




;pl1 : f1 channel - power level for pulse (default)
;pl3 : f3 channel - power level for pulse (default)
;pl12: f2 channel - power level for CPD/BB decoupling
;pl16: f3 channel - power level for CPD/BB decoupling in CON experiment
;pl26: f3 channel - power level for CPD/BB decoupling in HN experiment
;sp13: f1 channel - shaped pulse 180 degree  (adiabatic)
;sp2 : f1 channel - shaped pulse  90 degree  (on resonance)
;sp3 : f1 channel - shaped pulse 180 degree  (on resonance)
;sp5 : f1 channel - shaped pulse 180 degree  (Ca off resonance)
;sp8 : f1 channel - shaped pulse  90 degree  (on resonance)
;                   for time reversed pulse
;p8 : f1 channel - 180 degree shaped pulse for inversion (adiabatic)
;p13: f1 channel -  90 degree shaped pulse
;p14: f1 channel - 180 degree shaped pulse
;p16: homospoil/gradient pulse                        [1 msec]
;p21: f3 channel -  90 degree high power pulse
;p22: f3 channel - 180 degree high power pulse
;d32 : incremented delay (F1 in 2D)                    [3 usec]
;d1 : relaxation delay; 1-5 * T1
;d11: delay for disk I/O                              [30 msec]
;d12: delay for power switching                       [20 usec]
;d16: delay for homospoil/gradient recovery
;d22: 1/(4J(COCa))                                    [4.5 msec]
;d23: 1/(4J(NCO))                                     [12.5 msec]
;d60: relaxation delay exp1 (btHN)
;d59: acquisition exp1 (btHN)
;d58: acquistion exp2 (CACO)
;cnst21: CO chemical shift (offset, in ppm)
;cnst22: Calpha chemical shift (offset, in ppm)
;o1p: CO chemical shift (cnst21)
;l0: flag to switch between inphase and antiphase
;inf1: 1/SW(N) = 2 * DW(N)
;in32: 1/(2 * SW(N)) =  DW(N)
;nd32: 2
;ns: 8 * n
;ds: >= 32
;td1: number of experiments in F1 * 2
;FnMODE: States-TPPI (or TPPI) in F1
;cpd2: decoupling according to sequence defined by cpdprg2
;cpd3: decoupling according to sequence defined by cpdprg3
;pcpd2: f2 channel - 90 degree pulse for decoupling sequence
```

```
;pl1 : f1 channel - power level for pulse (default)
;pl3 : f3 channel - power level for pulse (default)
;pl16: f3 channel - power level for CPD/BB decoupling
;pl19: f1 channel - power level for CPD/BB decoupling
;sp2: f2 channel - shaped pulse  90 degree  (on resonance)
;sp3: f2 channel - shaped pulse 180 degree  (on resonance)
;sp5: f2 channel - shaped pulse 180 degree  (C=O off resonance)
;sp8: f2 channel - shaped pulse  90 degree  (on resonance)
;                  for time reversed pulse
;p1 : f1 channel -  90 degree high power pulse
;p2 : f1 channel - 180 degree high power pulse
;p13: f2 channel -  90 degree shaped pulse
;p14: f2 channel - 180 degree shaped pulse
;p16: homospoil/gradient pulse                        [1 msec]
;p21: f3 channel -  90 degree high power pulse
;p22: f3 channel - 180 degree high power pulse
;p26: f1 channel -  90 degree pulse at pl19
;d31 : incremented delay (F1 in 3D)                    [3 usec]
;d1 : relaxation delay; 1-5 * T1
;d3 : tau b : (2.2m-p14*2)/2
;d10: incremented delay (F2 in 3D) =  d23/2-p14/2
;d11: delay for disk I/O                              [30 msec]
;d12: delay for power switching                       [20 usec]
;d16: delay for homospoil/gradient recovery
;d23: T(N)                                            [12.4 msec]
;d22: tau d                                           [4.4 msec]
;d26: 1/(4J(NH)) - tau g                              [2.3 msec]
;d30: decremented delay (F2 in 3D) = d23/2-p14/2
;cnst21: CO chemical shift (offset, in ppm)
;cnst22: Calpha chemical shift (offset, in ppm)
;cnst23: Caliphatic chemical shift (offset, in ppm)
;cnst47: N(H) chemical shift (offset, in ppm)
;cnst57: N chemical shift (offset, in ppm)
;o2p: Caliphatic chemical shift (cnst23)
;inf1: 1/SW(Cali) = 2 * DW(Cali)
;in2: increment for f2 detection (15N all)
;in31: 1/(2 * SW(Cali)) =  DW(Cali)
;nd31: 2
;ns: 4 * n
;ds: >= 16
;td1: number of experiments in F1       td1 max = 2 * d41 / in41
;FnMODE: States-TPPI (or TPPI) in F1
;FnMODE: States-TPPI (or TPPI) in F2
;cpds1: decoupling according to sequence defined by cpdprg1
;cpd3: decoupling according to sequence defined by cpdprg3
;pcpd1: f1 channel - 90 degree pulse for decoupling sequence
;pcpd3: f3 channel - 90 degree pulse for decoupling sequence in CON experiment
;pcpd8: f3 channel - 90 degree pulse for decoupling sequence in HN experiment


;for z-only gradients:
;gpz11: 50%
;gpz12: 80%
;gpz13: 31%
;gpz14: 19%
;gpz15: 60%
;gpz16: 45%
;gpz17: 30.13%

;use gradient files:
;gpnam1: SMSQ10.100
;gpnam2: SMSQ10.100
```

```
;gpnam3: SMSQ10.100
;gpnam4: SMSQ10.100
;gpnam5: SMSQ10.100



;use AU-program splitcomb [ipap 2] to process data



;$Id: $
```

The mr_H^{αflip}CACO//btHN pulse sequence

```
;ut_bthn_hflipcaco.ms.t1
;avance-version (18/09/17)

;Dataset 1 (F1)
;best-TROSY
;2D H-1/X correlation via TROSY
;   using sensitivity improvement
;phase sensitive using Echo/Antiecho gradient selection
;using f3 - channel
;uncompensated version d25=d26
;with additional 180degree pulse on N-15
;(use parameterset B_TROSYETF3GPSI)
;
;A. Favier & B. Brutscher, J. Biomol. NMR 49, 9-15 (2011)
;(E. Lescop, P. Schanda & B. Brutscher,
;   J. Magn. Reson.  187 163-169 (2007))
;(T. Schulte-Herbrueggen & O.W. Sorensen, J. Magn. Reson. 144,
;   123 - 128 (2000))
;
;$CLASS=HighRes
;$DIM=2D
;$TYPE=
;$SUBTYPE=
;$COMMENT=

;Dataset 2 (F2)
;CON
;2D sequence with
;   13C detected correlation for triple resonance using
;      inept transfer steps
;
;      F1(C=O) -> F3(N,t1) -> F1(C=O,t2)
;
;on/off resonance 13C pulses using shaped pulses
;phase sensitive (t1)
;using IPAP scheme for virtual decoupling
;(use parameterset C_CON_IASQ)
;
;W. Bermel, I. Bertini,L. Duma, I.C. Felli, L. Emsley,  R. Pierattelli,
;   P.R. Vasos, Angew. Chem. Int. Ed. 44, 3089-3092 (2005)
;(L. Duma, S. Hediger, A. Lesage & L. Emsley,
;   J. Magn. Reson. 164, 187-195 (2003) )
;
;$CLASS=HighRes
;$DIM=2D
;$TYPE=
;$SUBTYPE=
;$COMMENT=


prosol relations=<triple>


#include <Avance.incl>
#include <Delay.incl>
#include <Grad.incl>


define list<gradient> EA3 = { 1.0000 0.8750 }
define list<gradient> EA5 = { 0.6667 1.0000 }
```

```
define list<gradient> EA7 = { 1.0000 0.6595 }

"p2=p1*2"
"p22=p21*2"

"p29=250" ;from bthn

"d11=30m"
"d12=20u"


"d1=(d60+d59)*2+d58" ;d60 recovery bthn d59 aq bthn d58 aq caco
"d3=1.1m"
"d4=1.8m-4u-p16-d16"
"d22=4.5m" ;from c_caco_ia
"d27=14.2m"

"d25=2.7m" ;from bthn
"d26=2.7m" ;from bthn

"d31=3u" ;from bthn
"d32=d22-d3+4u";from c_caco_ctia
"d33=d27-p14*1.5";from c_caco_ctia

"in31=inf1/2"  ;from bthn
"in32=in2/2"   ;from c_caco_ia
"in33=in2/2"    ;from c_caco_ctia




#   ifdef CALC_SP
"p42=(bwfac26/(cnst55*cnst52*bf1))*1000000"
"spw26=plw1*pow((p1*totrot26)/(p42*90.0*integfac26),2)"
"spoal26=0.5"

"p43=(bwfac28/(cnst55*cnst53*bf1))*1000000"
"spw28=plw1*pow((p1*totrot28)/(p43*90.0*integfac28),2)"
"spw29=plw1*pow((p1*totrot29)/(p43*90.0*integfac29),2)"
"spoal28=1"
"spoal29=0"
#   endif /*CALC_SP*/



"DELTA=d31*2+p8+p21*4/PI"
"DELTA1=d26-p19-d16-larger(p22,p42)/2"
"DELTA10=d25-p29-d16-larger(p22,p42)/2-p43*cnst43"
"DELTA11=d26-p16-d16-larger(p22,p42)/2"
"DELTA12=de+4u"


"DELTA2=p2+p18-p13"
"DELTA3=d3-p2/2"
"DELTA4=d3+p2/2"
"DELTA5=d22-d32-p1-p2"
"DELTA6=d27-d22-p14*1.5"



;"FACTOR1=d28*10000000/l3" ;from c_caco_ia
;"INCR1=FACTOR1/10000000" ;from c_caco_ia
```

```
;"if ( INCR1 > in32 ) { in33 = 0; } else { in33=in32-INCR1; }" ;from c_caco_ia
;"if ( INCR1 > in32 ) { in28 = in32; } else { in28 = INCR1; }" ;from c_caco_ia

"l0=1"

"l3=td1/2"


"spoffs2=0"
"spoffs3=0"
"spoffs4=bf2*((cnst23-cnst22)/1000000)"
"spoffs8=0"
"spoffs9=0"
"spoffs5=bf2*((cnst22-cnst21)/1000000)"
"spoffs20=bf2*((cnst21-cnst22)/1000000)"


"spoffs26=bf1*(cnst54/1000000)-o1"
"spoffs28=bf1*(cnst54/1000000)-o1"
"spoffs29=bf1*(cnst54/1000000)-o1"
"spoffs13=bf2*((cnst26-cnst21)/1000000)"



1 4u ze1 do:f1
  4u ze2
  d1

2 d11 do:f1 do:f3
  3m
3 12m do:f1 do:f3
4 6m do:f1 do:f3
5 12m do:f1 do:f3 ;d1

; bthn

6 d60
  20u fq=cnst21(bf ppm):f2
  d12 pl1:f1 pl3:f3
  50u UNBLKGRAD
  (p22 ph11):f3
  p16:gp3
  d16

  (p43:sp28 ph13)
  p19:gp11
  d16
  DELTA1
  (center (p42:sp26 ph12) (p22 ph11):f3 )
  DELTA1
  p19:gp11
  d16
  (p43:sp29 ph12):f1

  p16:gp12
  d16
  4u

  (p21 ph15):f3
  d31
```

```
  ;(p8:sp13 ph11):f2

  d31
  (p22 ph11):f3
  DELTA

  p16:gp13*EA3
  d16

  (p43:sp29 ph16)
  p29:gp14
  d16
  DELTA10
  (center (p42:sp26 ph12) (p22 ph12):f3 )
  DELTA10
  p29:gp14
  d16
  (p43:sp28 ph11)

  p16:gp15*EA5
  d16
  DELTA12

  (p21 ph11):f3
  p16:gp16
  d16
  DELTA11
  (center (p42:sp26 ph12) (p22 ph12):f3 )
  DELTA11
  p16:gp16
  d16 pl1:f1
  (p21 ph17:r):f3

  p16:gp17*EA7
  d16

  4u BLKGRAD

  goscnp1 ph31

  lo to 6 times 2

;c_caco

  8u do:f3 do:f1
  50u UNBLKGRAD
  ;20u pl19:f1
  20u pl1:f1
  20u pl16:f3
  20u fq=cnst22(bf ppm):f2
  d12 cpd3:f3 ;cpds1:f1

  p1 ph1
  d4
  4u
  p16:gp2
  d16
  (center (p18:sp4 ph1):f2 (p2 ph1):f1)
  4u
  p16:gp2
  d16
  d4
```

```
  p1 ph2

;  p16:gp2
;  d16

;  (p13:sp2 ph3):f2
;  d3 pl19:f1
;  d32 cpds1:f1

  DELTA2

  (p13:sp2 ph1):f2
  DELTA3
  p2 ph2
  (p18:sp4 ph1):f2 ; 180 ca medium selectivity @500 p16 888us Q3_surbop.1 SPOAL=0.5
  DELTA4
  p1 ph8

  d32
  p2 ph1
  DELTA5

  (p14:sp20 ph7):f2
  DELTA6
  (p14:sp3 ph1):f2
  d33
  (p14:sp20 ph1):f2
  4u
  (p13:sp8 ph4):f2

  p16:gp1
  d16 fq=cnst21(bf ppm):f2


  if "l0 %2 == 1"
     {
     (p13:sp2 ph5):f2
     4u
     (p14:sp5 ph1):f2
     d22
     (p14:sp3 ph1):f2
     4u
     (p14:sp5 ph1):f2
     d22 BLKGRAD ;do:f1
     }
  else
     {
     (p13:sp2 ph6):f2
     4u
     d22*0.5
     (p14:sp5 ph1):f2
     d22*0.5
     (p14:sp3 ph1):f2
     4u
     d22*0.5
     (p14:sp5 ph1):f2
     d22*0.5 BLKGRAD ;do:f1
     }

  go2=2 ph30 ;cpds1:f1

  d11 do:f1 do:f3 wr2 #1 if2 #1 zd2
```

```
;exp_f2 ipap
  3m iu0
  lo to 3 times 2

  3m wr1 #0 if1 #0 zd1

;exp_f1 phase
  3m ip16*2
  3m ip17*2
  3m ip15*2
  3m ip31*2

;exp_f1 gradients
  3m igrad EA3
  3m igrad EA5
  3m igrad EA7

;exp_f2 phase
  3m ip4
  lo to 4 times 2

;exp_f1 delay
  3m id31
;exp_f2 delay
  3m id32
  3m dd33
  lo to 5 times l3

exit


ph1=0
ph2=1
ph4=0 2
ph5=0 0 2 2
ph6=3 3 1 1
ph7=0 0 0 0 2 2 2 2
ph8=2

ph11=0
ph12=1
ph13=2
ph14=3
ph15=0 2
ph16=1
ph17=1
ph21=2


ph30=0 2 2 0
ph31=0 2 0 2




;pl1 : f1 channel - power level for pulse (default)
;pl3 : f3 channel - power level for pulse (default)
;pl12: f2 channel - power level for CPD/BB decoupling
;pl16: f3 channel - power level for CPD/BB decoupling in CON experiment
;pl26: f3 channel - power level for CPD/BB decoupling in HN experiment
```

```
;sp13: f1 channel - shaped pulse 180 degree  (adiabatic)
;sp2 : f1 channel - shaped pulse  90 degree  (on resonance)
;sp3 : f1 channel - shaped pulse 180 degree  (on resonance)
;sp4 : f2 channel - shaped pulse 180 degree Ca medium selectivity (on resonance)
;sp5 : f1 channel - shaped pulse 180 degree  (Ca off resonance)
;sp8 : f1 channel - shaped pulse  90 degree  (on resonance)
;                   for time reversed pulse
;p8 : f1 channel - 180 degree shaped pulse for inversion (adiabatic)
;p13: f1 channel -  90 degree shaped pulse
;p14: f1 channel - 180 degree shaped pulse
;p16: homospoil/gradient pulse                           [1 msec]
;p18: f2 channel - 180 degree med sel on Ca (888 us)
;p21: f3 channel -  90 degree high power pulse
;p22: f3 channel - 180 degree high power pulse
;d32 : incremented delay (F1 in 2D)                       [3 usec]
;d1 : relaxation delay; 1-5 * T1
;d11: delay for disk I/O                                 [30 msec]
;d12: delay for power switching                          [20 usec]
;d16: delay for homospoil/gradient recovery
;d22: 1/(4J(COCa))                                       [4.5 msec]
;d23: 1/(4J(NCO))                                        [12.5 msec]
;cnst21: CO chemical shift (offset, in ppm)
;cnst22: Calpha chemical shift (offset, in ppm)
;o1p: CO chemical shift (cnst21)
;l0: flag to switch between inphase and antiphase
;inf1: 1/SW(N) = 2 * DW(N)
;in32: 1/(2 * SW(N)) =  DW(N)
;nd32: 2
;ns: 8 * n
;ds: >= 32
;td1: number of experiments in F1 * 2
;FnMODE: States-TPPI (or TPPI) in F1
;cpd2: decoupling according to sequence defined by cpdprg2
;cpd3: decoupling according to sequence defined by cpdprg3
;pcpd2: f2 channel - 90 degree pulse for decoupling sequence

;pl1 : f1 channel - power level for pulse (default)
;pl3 : f3 channel - power level for pulse (default)
;pl16: f3 channel - power level for CPD/BB decoupling
;pl19: f1 channel - power level for CPD/BB decoupling
;sp2: f2 channel - shaped pulse  90 degree  (on resonance)
;sp3: f2 channel - shaped pulse 180 degree  (on resonance)
;sp5: f2 channel - shaped pulse 180 degree  (C=O off resonance)
;sp8: f2 channel - shaped pulse  90 degree  (on resonance)
;                  for time reversed pulse
;p1 : f1 channel -  90 degree high power pulse
;p2 : f1 channel - 180 degree high power pulse
;p13: f2 channel -  90 degree shaped pulse
;p14: f2 channel - 180 degree shaped pulse
;p16: homospoil/gradient pulse                           [1 msec]
;p21: f3 channel -  90 degree high power pulse
;p22: f3 channel - 180 degree high power pulse
;p26: f1 channel -  90 degree pulse at pl19
;d31 : incremented delay (F1 in 3D)                       [3 usec]
;d1 : relaxation delay; 1-5 * T1
;d3 : tau b : (2.2m-p14*2)/2
;d10: incremented delay (F2 in 3D) =  d23/2-p14/2
;d11: delay for disk I/O                                 [30 msec]
;d12: delay for power switching                          [20 usec]
;d16: delay for homospoil/gradient recovery
;d23: T(N)                                               [12.4 msec]
;d22: tau d                                              [4.4 msec]
```

```
;d26: 1/(4J(NH)) - tau g                              [2.3 msec]
;d30: decremented delay (F2 in 3D) = d23/2-p14/2
;cnst21: CO chemical shift (offset, in ppm)
;cnst22: Calpha chemical shift (offset, in ppm)
;cnst23: Caliphatic chemical shift (offset, in ppm)
;cnst47: N(H) chemical shift (offset, in ppm)
;cnst57: N chemical shift (offset, in ppm)
;o2p: Caliphatic chemical shift (cnst23)
;inf1: 1/SW(Cali) = 2 * DW(Cali)
;in2: increment for f2 detection (15N all)
;in31: 1/(2 * SW(Cali)) =  DW(Cali)
;nd31: 2
;ns: 4 * n
;ds: >= 16
;td1: number of experiments in F1       td1 max = 2 * d41 / in41
;FnMODE: States-TPPI (or TPPI) in F1
;FnMODE: States-TPPI (or TPPI) in F2
;cpds1: decoupling according to sequence defined by cpdprg1
;cpd3: decoupling according to sequence defined by cpdprg3
;pcpd1: f1 channel - 90 degree pulse for decoupling sequence
;pcpd3: f3 channel - 90 degree pulse for decoupling sequence in CON experiment
;pcpd8: f3 channel - 90 degree pulse for decoupling sequence in HN experiment


;for z-only gradients:
;gpz1: 50%
;gpz2: 80%
;gpz3: 31%
;gpz4: 19%
;gpz5: 60%

;use gradient files:
;gpnam1: SMSQ10.100
;gpnam2: SMSQ10.100
;gpnam3: SMSQ10.100
;gpnam4: SMSQ10.100
;gpnam5: SMSQ10.100



;use AU-program splitcomb [ipap 2] to process data



;$Id: $
```

References

1. Górka M, Żerko S, Konrat R, Koźmiński W, Kurzbach D. ^{1}H, ^{13}C and ^{15}N backbone resonance assignment of BRCA1 fragment 219–504. *Biomol NMR Assign.* 2020;14(2):289-293. doi:10.1007/s12104-020-09963-6

2. Somlyay M, Ledolter K, Kitzler M, Sandford G, Cobb SL, Konrat R. ^{19}F NMR Spectroscopy Tagging and Paramagnetic Relaxation Enhancement-Based Conformation Analysis of Intrinsically Disordered Protein Complexes. *ChemBioChem.* 2020;21(5):696-701. doi:10.1002/cbic.201900453

3. Felli IC, Pierattelli R. ^{13}C Direct Detected NMR for Challenging Systems. *Chem Rev.* 2022;122(10):9468-9496. doi:10.1021/acs.chemrev.1c00871

4. Pontoriero L, Schiavina M, Murrali MG, Pierattelli R, Felli IC. Monitoring the Interaction of α-Synuclein with Calcium Ions through Exclusively Heteronuclear Nuclear Magnetic Resonance Experiments. *Angew Chemie Int Ed.* 2020;59(42):18537-18545. doi:10.1002/anie.202008079

5. Grzesiek S, Bax A. Improved 3D triple-resonance NMR techniques applied to a 31 kDa protein. *J Magn Reson.* 1992;96(2):432-440. doi:10.1016/0022-2364(92)90099-S

6. Yamazaki T, Lee W, Arrowsmith CH, Muhandiram DR, Kay LE. A Suite of Triple Resonance NMR Experiments for the Backbone Assignment of ^{15}N, ^{13}C, ^{2}H Labeled Proteins with High Sensitivity. *J Am Chem Soc.* 1994;116(26):11655-11666. doi:10.1021/ja00105a005

7. Muhandiram DR, Kay LE. Gradient-Enhanced Triple-Resonance Three-Dimensional NMR Experiments with Improved Sensitivity. *J Magn Reson Ser B.* 1994;103(3):203-216. doi:10.1006/jmrb.1994.1032

8. Bracken C, Palmer AG, Cavanagh J. (H)N(COCA)NH and HN(COCA)NH experiments for ^{1}H-^{15}N backbone assignments in ^{13}C/^{15}N-labeled proteins. *J Biomol NMR.* 1997;9(1):94-100. doi:10.1023/a:1018679819693

9. Weisemann R, Rüterjans H, Bermel W. 3D Triple-resonance NMR techniques for the sequential assignment of N^{H} and ^{15}N resonances in ^{15}N- and ^{13}C-labelled proteins. *J Biomol NMR.* 1993;3(1). doi:10.1007/BF00242479

10. Bermel W, Bertini I, Felli IC, Kümmerle R, Pierattelli R. Novel ^{13}C direct detection experiments, including extension to the third dimension, to perform the complete assignment of proteins. *J Magn Reson.* 2006;178(1):56-64. doi:10.1016/j.jmr.2005.08.011

11. Lescop E, Schanda P, Brutscher B. A set of BEST triple-resonance experiments for time-optimized protein resonance assignment. *J Magn Reson.* 2007;187(1):163-169. doi:10.1016/j.jmr.2007.04.002

12. Solyom Z, Schwarten M, Geist L, Konrat R, Willbold D, Brutscher B. BEST-TROSY experiments for time-efficient sequential resonance assignment of large disordered proteins. *J Biomol NMR.* 2013;55:311-321. doi:10.1007/s10858-013-9715-0

13. Slad S, Bermel W, Kümmerle R, Mathieu D, Luy B. Band-selective universal 90° and 180° rotation pulses covering the aliphatic carbon chemical shift range for triple resonance experiments on 1.2 GHz spectrometers. *J Biomol NMR.* 2022;76(5-6):185-195. doi:10.1007/s10858-022-00404-1

14. Bohlen J-M, Rey M, Bodenhausen G. Refocusing with chirped pulses for broadband excitation without phase dispersion. *J Magn Reson.* 1989;84(1):191-197. doi:10.1016/0022-2364(89)90018-8

15. Klika KD. The Application of Simple and Easy to Implement Decoupling Pulse Scheme Combinations to Effect Decoupling of Large J Values with Reduced Artifacts. *Int J Spectrosc.* 2014;2014:1-9. doi:10.1155/2014/289638

16. Delaglio F, Grzesiek S, Vuister G, Zhu G, Pfeifer J, Bax A. NMRPipe: A multidimensional spectral processing system based on UNIX pipes. *J Biomol NMR.* 1995;6(3). doi:10.1007/BF00197809

17. Lee W, Rahimi M, Lee Y, Chiu A. POKY: a software suite for multidimensional NMR and 3D structure calculation of biomolecules. Gorodkin J, ed. *Bioinformatics.* 2021;37(18):3041-3042. doi:10.1093/bioinformatics/btab180
